# Deletion of Dicer in Smooth Muscle Affects Voiding Pattern and Reduces Detrusor Contractility and Neuroeffector Transmission

**DOI:** 10.1371/journal.pone.0035882

**Published:** 2012-04-27

**Authors:** Mardjaneh Karbalaei Sadegh, Mari Ekman, Catarina Rippe, Bengt Uvelius, Karl Swärd, Sebastian Albinsson

**Affiliations:** 1 Department of Experimental Medical Science, Biomedical Centre, Lund University, Lund, Sweden; 2 Department of Urology, Clinical Sciences, Lund University, Lund, Sweden; National Institutes of Health, United States of America

## Abstract

MicroRNAs have emerged as important regulators of smooth muscle phenotype and may play important roles in pathogenesis of various smooth muscle related disease states. The aim of this study was to investigate the role of miRNAs for urinary bladder function. We used an inducible and smooth muscle specific Dicer knockout (KO) mouse which resulted in significantly reduced levels of miRNAs, including miR-145, miR-143, miR-22, miR125b-5p and miR-27a, from detrusor preparations without mucosa. Deletion of Dicer resulted in a disturbed micturition pattern *in vivo* and reduced depolarization-induced pressure development in the isolated detrusor. Furthermore, electrical field stimulation revealed a decreased cholinergic but maintained purinergic component of neurogenic activation in Dicer KO bladder strips. The ultrastructure of detrusor smooth muscle cells was well maintained, and the density of nerve terminals was similar. Western blotting demonstrated reduced contents of calponin and desmin. Smooth muscle α-actin, SM22α and myocardin were unchanged. Activation of strips with exogenous agonists showed that depolarization-induced contraction was preferentially reduced; ATP- and calyculin A-induced contractions were unchanged. Quantitative real time PCR and western blotting demonstrated reduced expression of Cav1.2 (*Cacna1c*). It is concluded that smooth muscle miRNAs play an important role for detrusor contractility and voiding pattern of unrestrained mice. This is mediated in part via effects on expression of smooth muscle differentiation markers and L-type Ca^2+^ channels in the detrusor.

## Introduction

Emptying of the urinary bladder depends on coordinated contraction of the detrusor and relaxation of the urethra [1], [2]. Contraction follows the release of neurotransmitters from motor nerves that are dispersed in the muscle bundles [3]. This results in a rapid elevation of the sarcoplasmic Ca^2+^ concentration, activation of myosin light chain kinase, phosphorylation of myosin, and, after a brief delay, force development [4]. Acetylcholine is central among the transmitters released from neural varicosities in the detrusor, and the muscarinic G_q_-coupled M_3_ receptor is primarily responsible for cholinergic detrusor activation [5]. M_2_ receptors also contribute by inhibiting the formation of cyclic AMP [6]. In addition to muscarinic mechanisms, purinergic signalling plays a role [7], and the relative contribution of purinergic versus cholinergic excitation varies between species and in pathological situations. In humans, for example, the relative size of the muscarinic component of neurogenic activation decreases with age and in bladder disturbances [7], [8], [9], [10]; this so called “atropine resistance” is accompanied by an increase in the relative dependence on purinergic activation.

Recently, microRNAs have emerged as promising targets for therapeutic intervention in various disease states [11]–[14]. miRNAs are short non-coding RNAs that regulate protein expression and cellular function [15]. Mature miRNAs are generated from cleavage of pre-miRNAs by the endonuclease Dicer and are then incorporated into the RNA-induced silencing complex, which mediates degradation or translational repression/activation of the target mRNA. MiRNAs were recently identified to have an important role for vascular smooth muscle development and function by regulating phenotypic modulation, contractile function and neointimal hyperplasia [16]–[23]. Dramatic effects of miRNA depletion on smooth muscle differentiation and remodelling has also been reported for gastrointestinal smooth muscle [24]. In urinary bladder, a role of miRNAs in cancer has been identified [25]. However, the importance of miRNAs for detrusor smooth muscle function and phenotype modulation is unknown.

In this study we have used an inducible and smooth muscle specific Dicer knockout (KO) mouse to investigate the role of miRNAs for bladder function. We found that loss of miRNAs resulted in a decreased contractile function in response to depolarization, which was associated with reduced expression of contractile marker proteins and L-type Ca^2+^ channels. Our findings also imply that miRNAs in smooth muscle may play a role for cholinergic neuro-effector transmission in the urinary bladder.

## Materials and Methods

### Animals

Adult mice with inducible and smooth muscle cell specific inactivation of Dicer were generated as described previously [26]. At the age of 4 weeks, male SMMHC-CreERT2/Dicer^flox/flox^ (SM-Dicer KO) mice were treated with intraperitoneal injections of 0.1 ml Tamoxifen (50 mg/kg/day) or vehicle (1∶10 EtOH in sunflower oil) for 5 consecutive days. Vehicle treated male littermate mice were used as controls. Experiments were performed 5 or 10 weeks post tamoxifen injections. Mice were on a mixed C57Bl/6;129 background and all animal experiments were approved by the Lund/Malmö Ethics Committee (M167-09).

### MiRNA Arrays and Quantitative RT-PCR

Mice were sacrificed by increasing CO_2_ and whole bladders were excised and cleaned in HEPES buffered Krebs solution (nominally Ca^2+^-free). Bladders were cut open from the urethra and pinned to the bottom of Sylgard-covered dissection dishes containing physiological buffer. The mucosa was removed by pulling using fine forceps in combination with sharp micro-dissection. Following freezing in liquid N_2_, isolation of mRNA and miRNA from six pooled control and Dicer KO bladders without mucosa was performed using miRNeasy kit and RNeasy MinElute Cleanup Kit (Qiagen) according to the manufacturer’s recommendations. Whole genome, qPCR based, miRNA arrays (RT2 miRNA PCR Array mouse, #MAM-200C-2, SA Biosciences) were used according to the manufacturer’s instructions. Individual miRNAs and mRNAs were analyzed using miScript primer assays (Qiagen) and QuantiTect Primer assays (Qiagen), respectively.

### Voiding Patterns of Freely Moving Mice

Mice were housed individually in standard cages and 24 h urine output was collected on filter papers covering the whole cage area [27]. Papers were photographed under ultraviolet light. Spots were analyzed by blinded counting of the total number of spots, the number of spots bordering the edge of the filter paper (edge), and those that did not touch the edge of the paper (center).

### Isolated Bladder Preparation

The ureters of isolated bladders were ligated and the bladder was catheterized via the urethra. The catheter was connected to a pressure transducer (Living Systems Instrumentation) and a peristaltic pump. The bladder was then positioned in a 50 ml water jacketed bath containing aerated HEPES buffered Krebs solution (2.5 mM Ca^2+^, 37°C). Intravesicular pressure was continuously recorded using the PM4 perfusion pressure monitor (Living Systems Instrumentation) and WinDaq waveform recording software (Dataq Instruments). Buffer was injected through the catheter (50, 100 and 200 µl) in a stepwise fashion. After stabilization of passive pressure at each volume, 60 mM K^+^ (obtained by exchange of NaCl for KCl) was added to the bathing solution. Active pressure was maintained for 5 min and integrated over the entire stimulation period.

### Strip Preparations and Length-tension Relationship

Force measurements were done essentially as described [28] using equatorial bladder strips without mucosa. In brief, preparations were mounted in myographs with open organ baths (three 610 M, Danish MyoTechnology, Aarhus, Denmark) filled with aerated HEPES buffered Krebs solution (2.5 mM Ca^2+^, 37°C). Length was systematically increased and strips were contracted with 60 mM K^+^ at each length followed by relaxation in Ca^2+^-free HEPES buffered Krebs solution. The preparations were then stretched to a new length and allowed to equilibrate in Ca^2+^-containing buffer prior to contraction with 60 mM K^+^.

Electrical field stimulation, carbachol concentration-response relationships, and ATP responses were recorded at the optimal length for force development (L_0_). After each experiment the lengths and weights of the individual preparations were determined to allow for calculation of stress (force per cross-sectional area).

### Electrical Field Stimulation

Bladder smooth muscle strips without mucosa were prepared and mounted as described [29]. Full frequency response curves (5 s activation, pulse duration 0.5 ms, at 2 min intervals) were generated in control conditions, in the presence of scopolamine (1 µM), and after desensitization of purine receptors using α,β-methylene-ATP (10 µM) in the continued presence of scopolamine [28]. Each experiment was started and ended by depolarizing the smooth muscle with 125 mM KCl.

### Electron Microscopy

All bladders were filled with 0.3 ml saline through the urethra [21]. Processing for fixation and electron microscopy was performed as described [28]. 100 digital micrographs at three levels of magnification (10, 30, and 60 K) were acquired (600 in total) and analyzed using ImageJ (NIH, Bethesda, MD, USA).

### Western Blotting

Detrusor muscle homogenates were prepared from control and Dicer KO bladders as described previously [28]. Briefly, the samples were frozen in liquid N_2_, dissolved in Laemmli buffer containing phosphatase and protease inhibitor cocktails (Bio-Rad). Following determination of protein concentration, equal amounts of protein were loaded on TGX Criterion gels (Biorad). Proteins were then transferred using either wet transfer over-night or semi-dry transfer for 10 min using the Trans-Blot Turbo system (Biorad). Proteins were detected using commercially available primary antibodies: Desmin (Cell Signaling, 1∶1000), Calponin (1∶1000) and SM22 (1∶2000) and Myocardin (Abcam, 1∶500), α-actin (Sigma, 1∶1000), HSP90 (BD Transduction labs., 1∶1000), Ca^2+^/calmodulin-dependent protein kinase (CamKIIδ, R&D Systems, 1∶500), and Cav1.2 (Alomone labs, 1∶500). HRP-conjugated or fluorescently labeled DyLight800 and DyLight680 secondary antibodies (Cell Signaling, 1∶5000) were used and images were acquired using the LI-COR Odyssey Fc instrument (LI-COR Biosciences).

### Statistical Analysis

All data are presented as means ± SEM and single comparisons between two groups were performed using student’s t-test. Multiple comparisons were performed using ANOVA followed by the Bonferroni post-hoc test. n≥3 for all experiments. * = p<0.05, ** = p<0.01, *** = p<0.001.

## Results

### Deletion of Dicer Results in a General Loss of miRNAs in the Bladder

**Figure 1:**
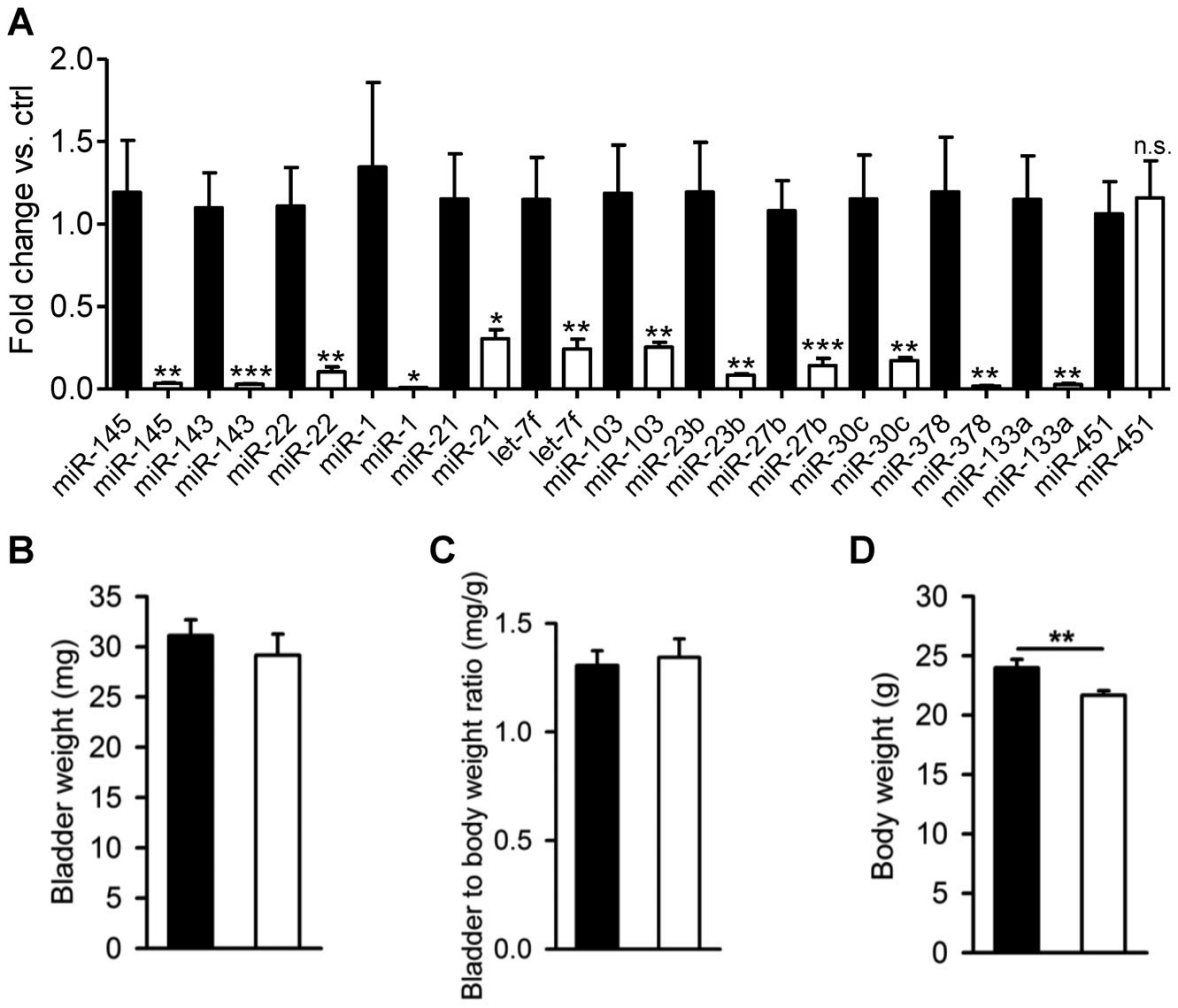
Reduced levels of detrusor miRNAs and maintained wet weight in Dicer KO urinary bladder. Highly expressed miRNAs were selected in an array experiment and analyzed here by qPCR in control (black bars) and Dicer KO (white bars) urinary bladders excised 10 weeks following tamoxifen treatment (A) (n = 6). MiR-451 is generated in Dicer independent manner and was included as a negative control. The bladder wet weights, bladder to body weight ratios, and body weights of control and Dicer KO bladders is shown in B-D (n = 13).

To assess the effect of smooth muscle specific Dicer knockout (KO) on the miRNA expression pattern in the urinary bladder, miRNA arrays were run using pooled samples from control and KO bladders without mucosa. MiR-145, miR-22, miR-125b-5p, miR-27a and miR-1 appeared to be most highly expressed in the bladder muscle layer, and their knockdown level ranged between 68 and 99% (Table S1). We chose 11 highly expressed miRNAs from the array for validation by quantitative RT-PCR. MiR-143 was not on the array but was included because it is generated together with miR-145 from a bicistronic transcript [16]. MiR-451 was included as a negative control as it is known to be generated in a Dicer independent manner [30], [31]. With the exception for miR-451, Dicer deletion resulted in significant reduction of all of these miRNAs (Figure 1A, 10 weeks post tamoxifen). Taken together, this demonstrates efficient knockdown of miRNAs in detrusor smooth muscle following deletion of Dicer.

Loss of miRNAs did not significantly affect bladder weight (Figure 1B) or bladder to body weight ratio (Figure 1C). As reported previously [32], the body weight was reduced 10 weeks following tamoxifen treatment (Figure 1D).

### Altered Micturition Pattern in the Absence of Smooth Muscle miRNAs

**Figure 2:**
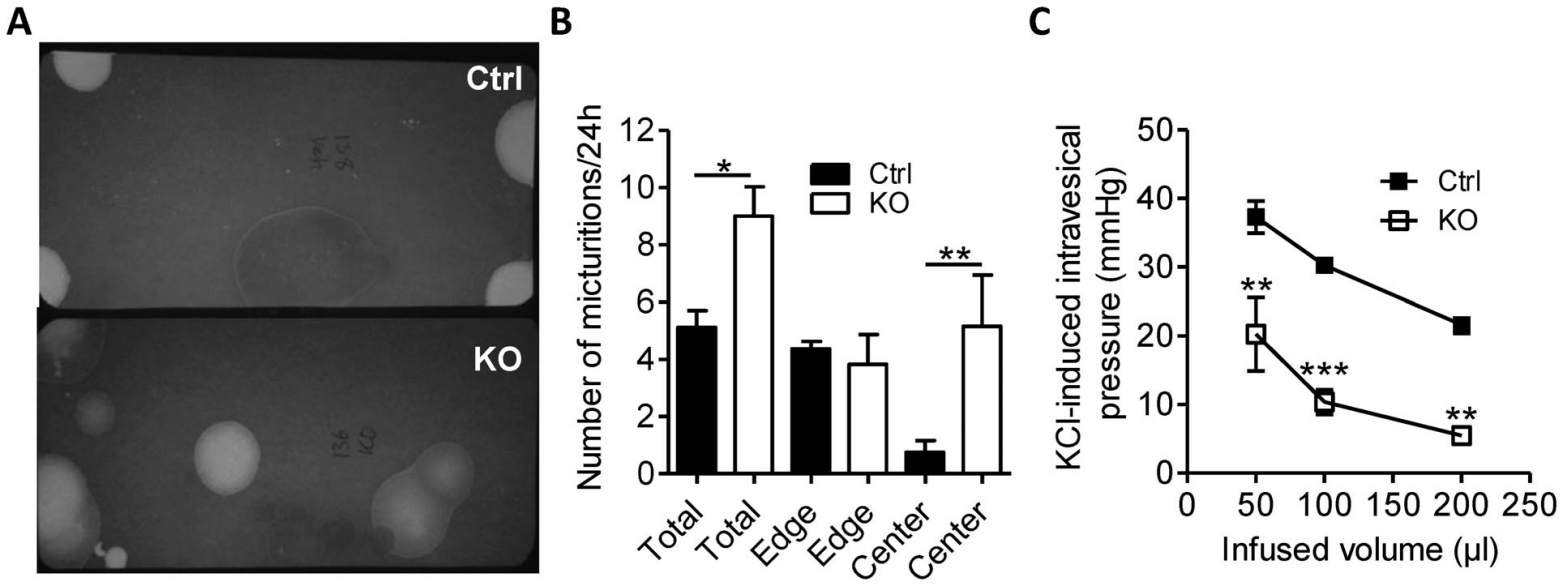
Disturbed micturition pattern and impaired pressure generation in the isolated bladder. Voided urine was collected on filter papers. Panel A shows filter papers photographed under UV light. Panel B shows summarized data. Panel C shows pressure of isolated and cannulated bladders during 5 min of stimulation with 60 mM K^+^ at different volumes (6–8).

To test if the loss of miRNAs affected the voiding pattern of freely moving mice, urine was collected on filter papers that were subsequently photographed under UV light (Figure 2A). Analysis of the number of spots demonstrated an increased number of spots for the KO compared to the control mice (Figure 2B). Mice tend to void in the corners or along the edge of the cage and this pattern changes in micturition disturbances [27]. In fact, the increased micturition frequency was accounted for by the increase in the centrally localized spots (Figure 2B). To address whether motor function of the KO detrusor was altered, bladders were fitted with pressure transducers. Bladder volume was then increased in a step-wise fashion to 200 µl. In resting bladders the pressure changed little with filling and no spontaneously generated pressure peaks were observed. Depolarization (60 mM K^+^) resulted in prompt increases in pressure. The amplitude of this response declined with increasing volume as expected from the law of Laplace. The attained pressure on activation with K^+^ was lower in KO bladders at all filling volumes (Figure 2C).

**Figure 3:**
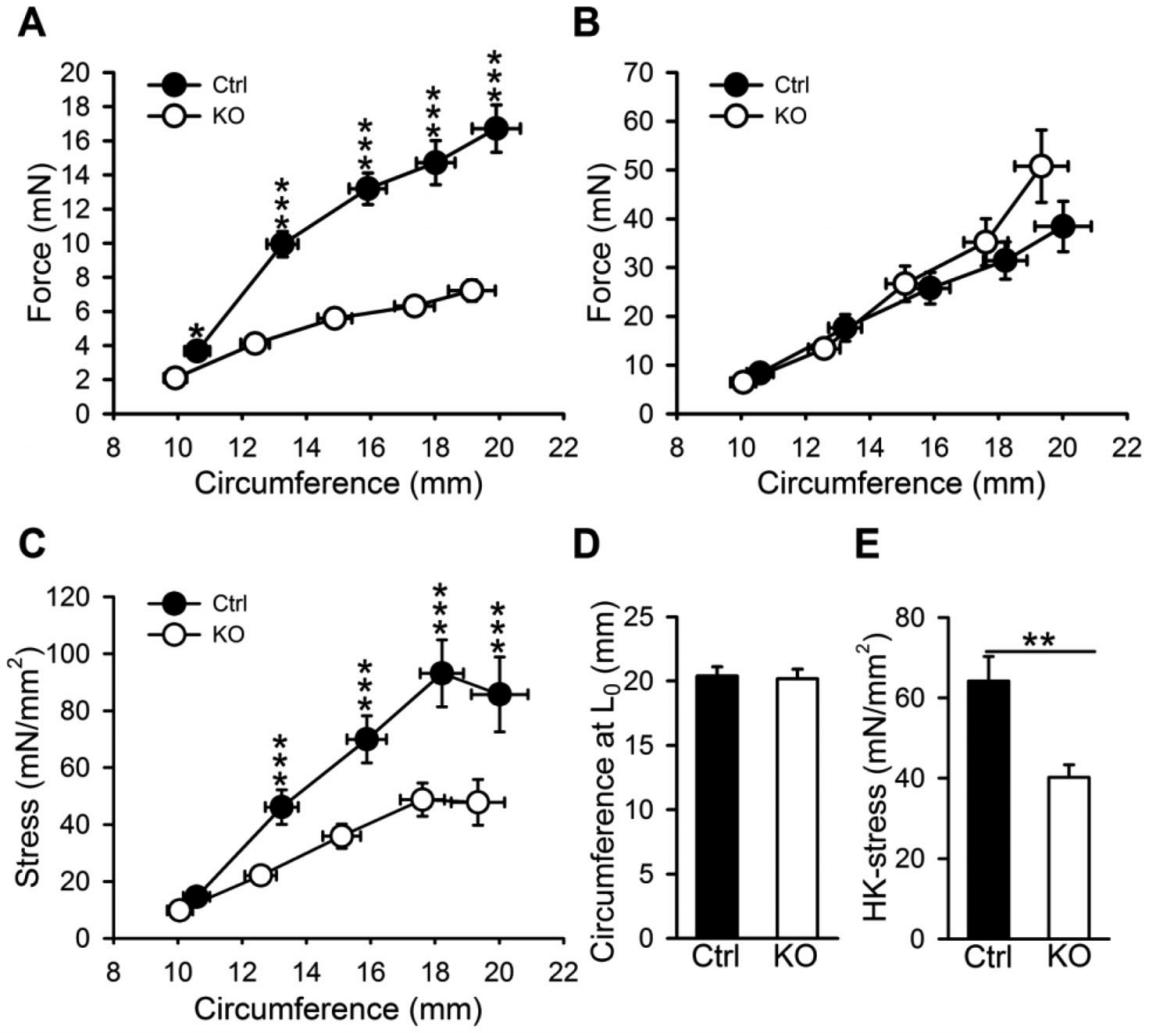
Reduced active stress in Dicer KO detrusor strips. Active (A) and passive (B) length-tension relationships were generated using strips from control and Dicer KO bladders. The muscle strips were stimulated in 60 mM K^+^ and relaxed in nominally calcium-free solution. Stress (C) was calculated using the length and weight of the individual preparations. The calculated circumference at which the bladder generated maximal active force (L_0_) was not different in Dicer KO bladders (D). Stress at L_0_ is shown in E (n = 11).

### Detrusor Muscle Contractile Function is Impaired in the Absence of miRNAs

To confirm that the reduced ability of whole bladder preparations to generate pressure was due to impaired contractility we mounted strips of bladder smooth muscle in myographs and generated length-tension relationships. As shown in Figure 3A, active force in response to depolarization with 60 mM K^+^ was reduced in Dicer KO strips at all muscle lengths. Passive force on the other hand was not altered (Figure 3B). Stress (force per cross-sectional area) was reduced at circumferences exceeding 12 mm (Figure 3C). The optimal length for force development (L_0_) was not changed (Figure 3D), whereas force at L_0_ was reduced (Figure 3E). Taken together these findings confirm reduced contractility in the absence of detrusor miRNAs and additionally show that this occurs without remodeling.

**Figure 4:**
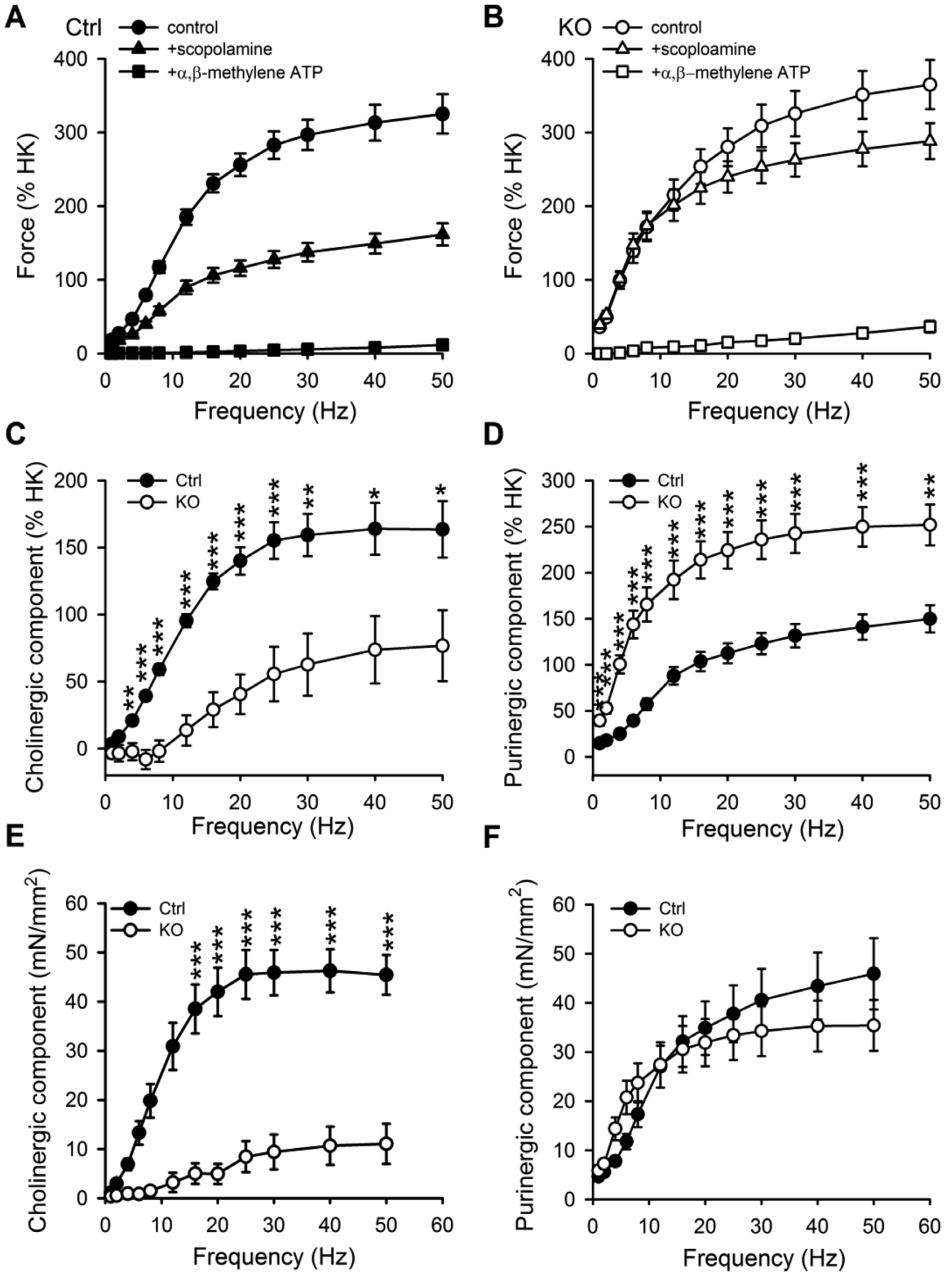
Deletion of Dicer in smooth muscle impairs the cholinergic component of the neurogenic contraction. Detrusor muscle strips from control and Dicer KO mice were activated by electrical field stimulation in the absence and presence of scopolamine and after desensitization of purinergic receptors using α,β-methylene-ATP in the continued presence of scopolamine. Full frequency response curves in control conditions, in the presence of scopolamine (1 µM), and after desensitization of purinergic receptors using α,β-methylene-ATP (10 µM) in the continued presence of scopolamine are shown for control and Dicer KO bladders in A and B, respectively, 10 weeks following Tamoxifen treatment. The cholinergic component of activation (C) was calculated by subtracting the force in the presence of scopolamine from force in control conditions. The purinergic component of activation (D) was calculated by subtracting residual force (after α,β-methylene-ATP and in the presence of scopolamine) from force in the presence of scopolamine. The stress (force per cross-sectional area) for the different components was calculated using absolute force values, strip length, strip weight, and assuming a density of 1.06; the resulting data is shown in E-F. Stress was then calculated for the scopolamine sensitive (cholinergic, E) and the α,β-methylene-ATP sensitive (purinergic, F) components (n = 10).

### Altered Responsiveness to Electrical Field Stimulation in Dicer KO Detrusor Muscle

Electrical field stimulation *ex vivo* causes release of neurotransmitter substances from autonomic nerves and subsequent muscle activation. Full frequency response relationships were generated in control and KO detrusor strips under control conditions, after inhibition of muscarinic receptors using scopolamine and after desensitization of purine receptors using α,β-methylene-ATP (Figure 4A-4D; c.f. Figure S1 for a merged version of 4A and 4B). We plotted the scopolamine-sensitive (cholinergic) and α,β-methylene-ATP-sensitive (purinergic) components of activation for control and Dicer KO bladders as stress (Figure 4E and 4F). A striking reduction of the muscarinic component was seen in Dicer KO compared to control detrusor. The purinergic component of activation on the other hand was unchanged. When force was normalized to K^+^ contraction (or maximal nerve-induced activation) rather than being expressed as stress, the cholinergic component remained reduced whereas the purinergic component was increased (Figure 4C and 4D). A similar, albeit less pronounced, increase of the relative purinergic component was evident already 5 w post Tamoxifen (Figure S2). Taken together, a state of “scopolamine resistance” is evident in the Dicer KO detrusor together with a relative increase of the purinergic component of neurogenic activation, i.e. changes known to occur in many pathological bladder disturbances.

**Figure 5:**
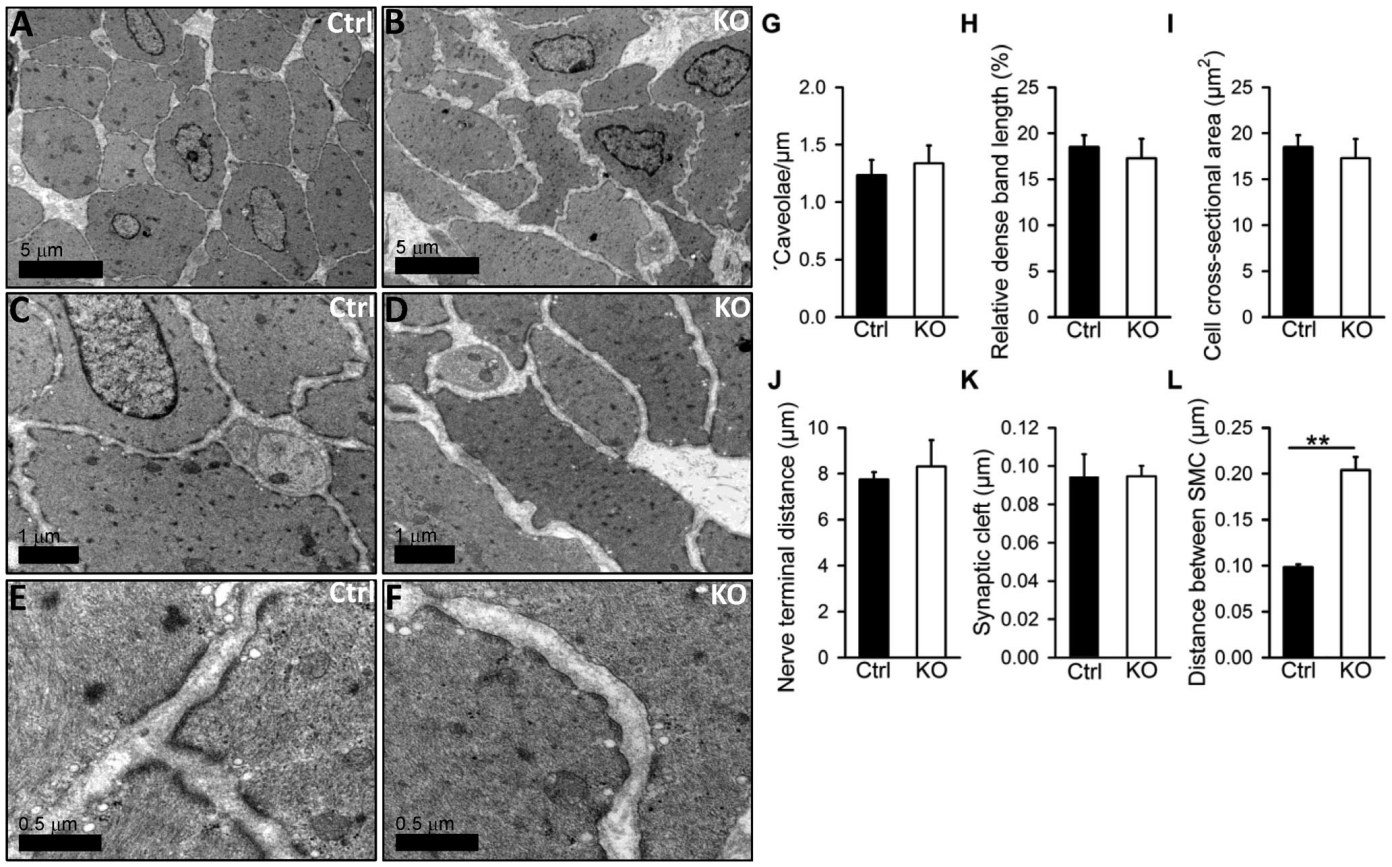
Electron microscopy reveals normal cell morphology and innervation but increased distance between cells in Dicer KO urinary bladder. Smooth muscle cells in control (A, C, E) and Dicer KO (B, D, F) bladders were analyzed by electron microscopy. 600 EM micrographs in total were used for quantitative analysis of the density of caveolae (G), the relative proportion of each cell profile that was occupied by dense bands (H), the cell cross sectional area (I), the distance between nerve terminals (J), the width of the synaptic cleft at sites with no Schwann cell coating (K), and the cell to cell distance (L) (n = 3–4).

**Figure 6:**
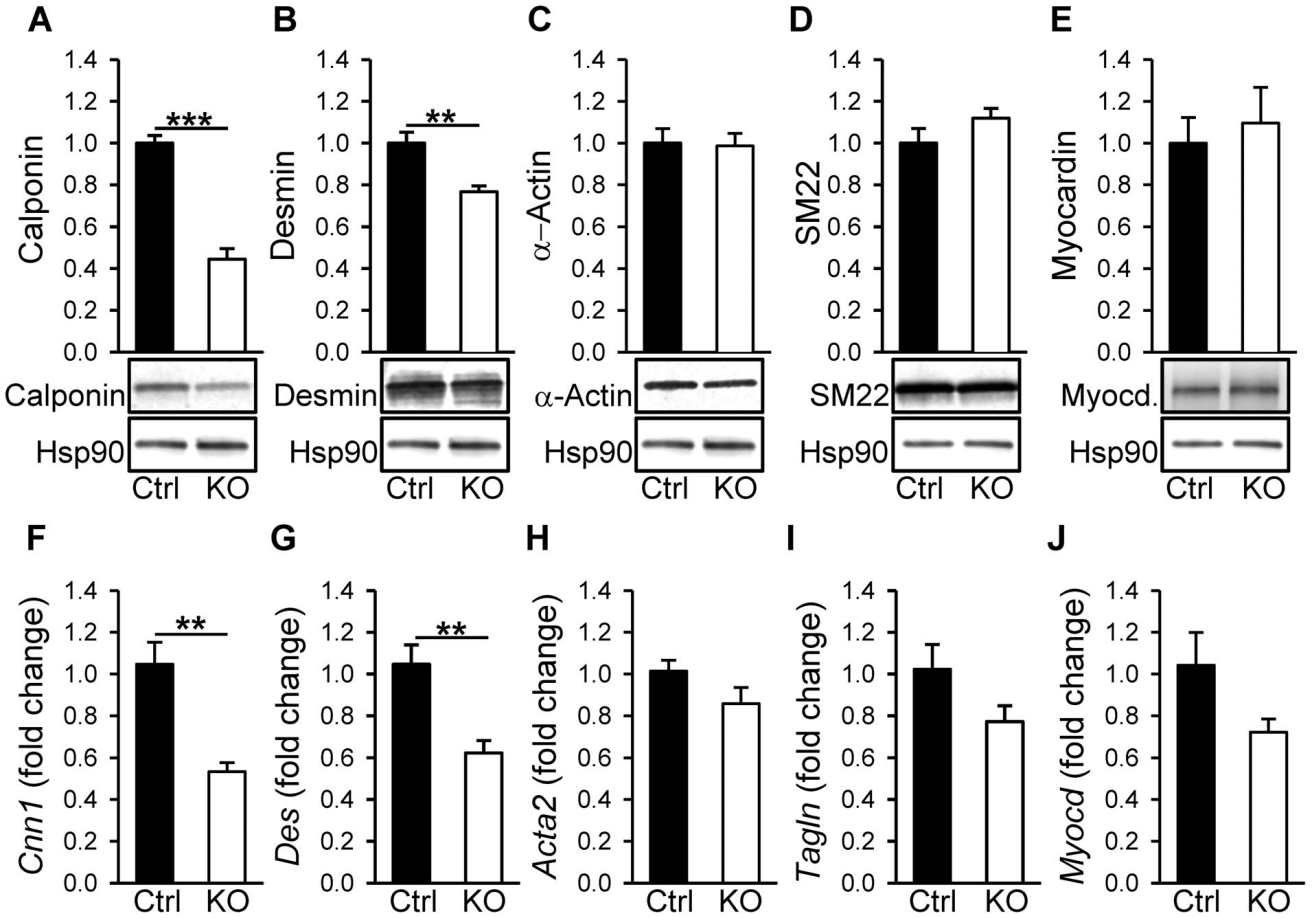
Reduced contents of calponin and desmin in Dicer KO detrusor. Expression of the differentiation related proteins calponin (A), desmin (B), smooth muscle α-actin (C), SM22 (D), and myocardin (E) was analyzed in control (black bars) and Dicer KO (white bars) bladders by western blotting 10 weeks post tamoxifen treatment. Original blots are shown below the individual bar graphs. HSP90 was used as loading control throughout (n = 6–8). Transcript levels for selected genes were examined in control and Dicer KO detrusor by qPCR 10 weeks post tamoxifen treatment. Primers for genes encoding calponin (*Cnn1*, F), Desmin (*Des*, G), smooth muscle α-actin (*Acta2*, H), SM22 (*Tagln*, I), Myocardin (*Myocd*, J) were used (n = 5–11).

### Electron Microscopy Analysis of Smooth Muscle Cells in Dicer KO Bladder

In order to examine if the disturbed contractility was associated with ultrastructural changes, detrusor smooth muscle was examined using electron microscopy (Figure 5A-5F). We used three control and three KO detrusors fixed at identical volumes to measure cell cross-sectional area, the percentage of the cell membrane length that was occupied by dense bands, the density of caveolae, the nerve terminal distances, the size of the synaptic clefts, and the distance between cells (Figure 5G-5L). Overall, the ultrastructure was well maintained in KO bladders. Except for an increase in the distance between smooth muscle cells, all other parameters remained similar in KO and control bladders (Figure 5G-5L). Thus, the reduced contractility in response to nerve activation occurs without a change in the density of neurons reaching the bladder and without conspicuous changes in the overall morphology or cross-sectional area of smooth muscle cells.

**Figure 7:**
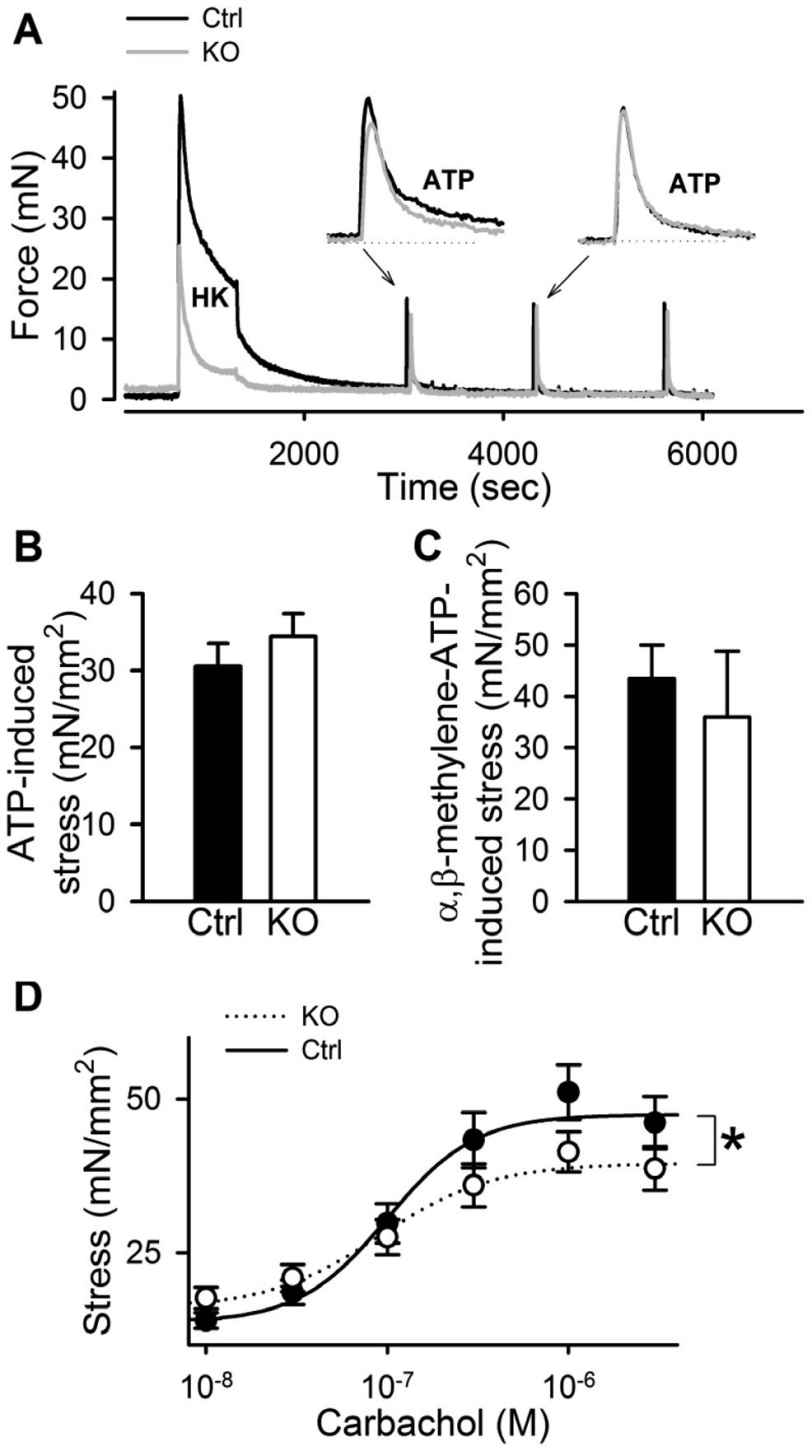
Selective reduction of depolarization-induced stress in Dicer KO bladder. Panel A shows original force records of control (black line) and Dicer KO (gray line) bladder strips. Following contraction in response to 60 mM K^+^ (HK) and relaxation, 3 mM ATP was added every 10 min. The preparations were washed three times following each ATP challenge. Insets show representative ATP responses on an expanded time scale (n = 4–7). B and C show summarized data for the peak ATP- and α,β-methylene-ATP-induced stress in control (black bar) and Dicer KO (white bar) bladders. Panel D shows cumulative concentration-response relationship for carbachol for control and Dicer KO bladders (n = 10–11).

**Figure 8:**
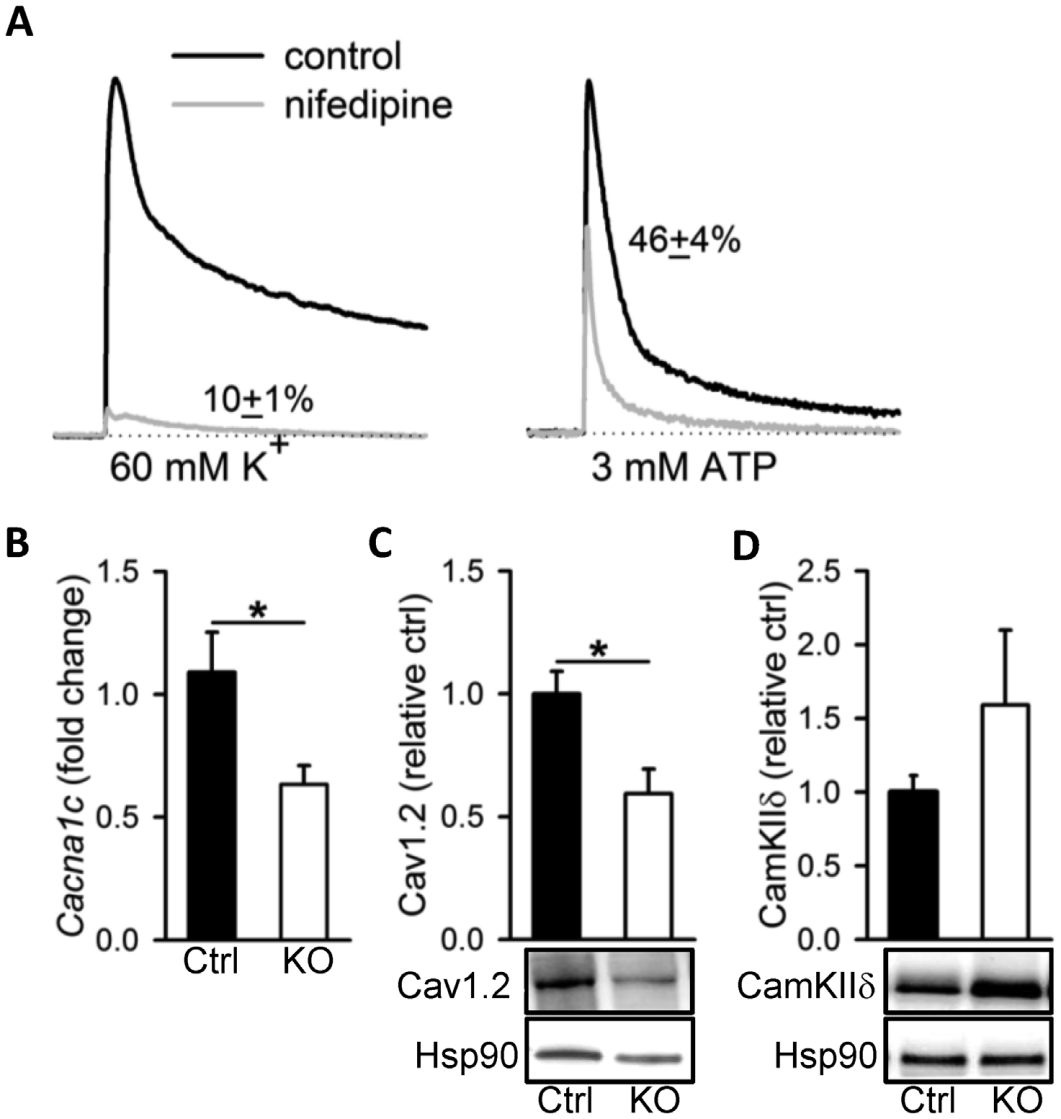
Reduced expression of L-type Ca^2+^ channels in Dicer KO bladder. Panel A shows contractile responses to 60 mM K^+^ and 3 mM ATP, respectively, before (black trace) and after (gray trace) addition of the L-type Ca^2+^ channel blocker nifedipine (1 µM). Both K^+^ (not shown) and ATP (Figure 7A) responses were highly reproducible (n = 4). Panels B and C show the mRNA and protein levels for the pore-forming subunit of the L-type Ca^2+^ channel (*Cacna1c* and Cav1.2) in control (black bars) and Dicer KO (white bars) bladder. Expression of CamKIIδ is shown in D (n = 6–10).

### Phenotypic Modulation of Detrusor Smooth Muscle in the Absence of miRNAs

In vascular smooth muscle, deletion of Dicer results in a general reduction of differentiation as characterized by a lower expression of smooth muscle differentiation markers. To examine if this occurs in the detrusor upon deletion of Dicer we determined the expression of calponin, desmin, α-actin, and SM22 by western blotting (Figure 6A-6E). Clear-cut reductions were seen in calponin and desmin whereas the levels of α-actin and SM22 were unchanged. Myocardin levels were similarly unchanged (Figure 6E). Quantitative RT PCR results mirrored the changes at the protein level and additionally showed a modest reduction of myosin heavy chain (*Myh11*) but unchanged *SRF* expression (Figure 6F-6J and data not shown). These findings indicate reduced expression of two contractile marker proteins, but they do not readily explain the selective reduction of depolarization-induced stress (Figure 3) relative to the purinergic component of nerve-induced stress (Figure 4F).

### Response of Dicer KO Detrusor Strips to Exogenous Agonists

In order to confirm that depolarization-induced contraction is indeed selectively impaired in the Dicer KO detrusor we contracted bladder strips with 60 mM K^+^. After washout and relaxation preparations were contracted with exogenous ATP (3 mM, Figure 7A). As predicted from the results in Figure 3 and 4, the depolarization-induced response was drastically reduced in comparison to the peak of the subsequent ATP response (p<0.001). ATP-induced stress on the other hand was unchanged (Figure 7B). Similar results were obtained for α,β-methylene-ATP (Figure 7C). We next generated full concentration-response curves for the muscarinic agonist carbachol. A modest reduction of stress was seen at saturating concentrations of carbachol (Figure 7D). However, carbachol contraction was increased following normalization to K^+^ contraction in the same strip (p<0.01, not shown), contrasting with the cholinergic neurogenic component. These findings verify a selective reduction of depolarization-induced contractility not only relative to ATP, but also relative to carbachol. In further support of this specificity we found that direct activation of the contractile machinery using the phosphatase inhibitor calyculin A resulted in similar contraction in control and Dicer KO detrusor strips (70±10 vs. 63±16 mN/mm^2^, n.s.).

### Reduced Expression of L-type Ca^2+^ Channels in Dicer KO Detrusor

Genetic ablation of L-type Ca^2+^ channels leads to selective impairment of KCl-induced contraction as compared to stimulation with carbachol (∼90% vs. ∼35% inhibition of tonic phase, [33]). Moreover, P_2_X_1_ channels, which are involved in detrusor activation by ATP, are directly permeable to Ca^2+^ which implies a less critical role of L-type channels in the ATP response compared to the KCl response. To test the validity of this concept under our assay conditions we pre-incubated bladder strips with the L-type Ca^2+^-channel blocker nifedipine (1 µM) and then contracted the strips with either 60 mM KCl or 3 mM ATP. As shown in Figure 8A, nifedipine inhibited KCl-induced contraction by 90±1%; ATP-induced contraction was inhibited by only 54±4% (p<0.001 vs. KCl). Reduced expression of L-type Ca^2+^-channels in Dicer KO bladder could thus potentially explain the specificity of miRNA deletion for KCl-induced contraction. We therefore assayed the level of transcript for the pore-forming subunit of the L-type Ca^2+^-channel (*cacna1c*) and the level of protein (Cav1.2). Both were reduced in Dicer KO detrusor (Figure 8B and 8C). Taken together, these findings argue that impaired motor function of the Dicer KO detrusor can be traced back to a reduced expression of L-type Ca^2+^ channels.

Recent work has indicated that *cacna1c*/Cav1.2 expression is negatively regulated by CamKIIδ activity [34], [35]. Because CamKIIδ is a predicted and validated target for the highly expressed miR-145 [16], we examined the expression of CamKIIδ by western blotting. The CamKIIδ level tended to be increased in KO detrusor, but this difference did not reach statistical significance (Figure 8D).

## Discussion

In the present study we used smooth muscle-specific and Tamoxifen-inducible knockout of Dicer to evaluate the importance of miRNAs for urinary bladder function in mice. We found that loss of miRNAs affected the voiding pattern of un-anaesthetized and freely moving animals and impaired the motor function of the intact bladder. These effects were associated with reduced contractile differentiation of the detrusor muscle and reduced expression of L-type Ca^2+^ channels. Our findings also imply that cholinergic neuro-effector transmission must be affected at a junctional or pre-junctional level. This is because the cholinergic component of neural activation was more drastically affected than the response to exogenous carbachol (reduced and increased relative to K^+^, respectively).

The effect of Dicer KO on detrusor smooth muscle marker expression is milder than previously reported by us for Dicer-deficient vascular smooth muscle [26], [32]. In vascular smooth muscle the effects are due in part to miR-145, which regulates smooth muscle differentiation via multiple mechanisms including myocardin expression [19], actin polymerization [18], [32], and angiotensin signaling [17]. Accordingly, robust reductions of smooth muscle α-actin, SM22, calponin and myosin heavy chain were previously found in Dicer-deficient vascular smooth muscle [26]. Here we find reductions of calponin and desmin, whereas the levels of smooth muscle α-actin, SM22 and myocardin were unchanged. Findings in the literature nonetheless support the idea that the changes that we observe may contribute to the contractile deficit. Genetic ablation of smooth muscle calponin, which is an actin binding protein that regulates actin-myosin interaction [36], was found to result in a 40% reduction of depolarization-induced force in aorta and vas deferens [37], [38]. Lack of the desmin similarly resulted in a 50% reduction of depolarization-induced stress in the urinary bladder [39].

A cause and effect relationship between the reduced levels of calponin and desmin and the impaired detrusor contractility is challenged by a number of observations. The first and most important observation was that Dicer deletion showed marked specificity for depolarization-induced force. Indeed, carbachol and ATP-induced contractions were much less affected or largely unaffected. When the membrane activation step was bypassed using the potent phosphatase inhibitor Calyculin A, no difference was observed between control and Dicer KO bladder, arguing for a largely functional contractile machinery. The ultrastructure of the smooth muscle moreover appeared normal, with normal cross-sectional areas of the muscle cells, thin and thick filaments, and no expansion of rough endoplasmic reticulum; that is, no hallmarks of the so called synthetic phenotype were evident. Taken together, this argues for additional effects of Dicer deletion at the level of membrane excitation.

Detrusor contraction shows a high degree of sensitivity to L-type Ca^2+^ channel blockers [40]. Here, we found that expression of the pore-forming subunit of the L-type Ca^2+^ channel (Cav1.2, *Cacna1c*) was reduced by ∼50% in Dicer KO detrusor. Work on smooth muscle-specific Cav1.2 knockout mice demonstrated that lack of these channels resulted in a ∼90% reduction of the tonic KCl response in detrusor strips. The tonic carbachol response on the other hand was reduced by only ∼35% [33]. Reduced expression Cav1.2 thus represents a likely reason for the preferential effect of Dicer deletion on KCl-induced contraction, and our finding that ATP responses were more resistant to nifedipine than were KCl responses concurs with this view. Previous work in smooth muscle has demonstrated reduced expression of L-type Ca^2+^ channels in phenotypically modulated smooth muscle cells [41]. Our own work on gastro-intestinal smooth muscle indicated that organ culture reduced L-type Ca^2+^ current in a Ca^2+^-dependent manner [42]. Since L-type Ca^2+^ channels directly contribute to the differentiation process [43], [44], their reduced expression may constitute an initial and critical step in the phenotype switch that is initiated by deletion of miRNAs from smooth muscle. The differential loss of smooth muscle marker expression in detrusor versus vascular smooth muscle at 10 weeks following Dicer deletion may therefore reflect different time-courses of L-type calcium channel expression in these tissues.

The basis of the reduced expression of L-type Ca^2+^ channels in Dicer KO bladder is not known. Recent work on cardiomyocytes has shown that overexpression and deletion, respectively, of CamKIIδ results in reduced and increased L-type Ca^2+^ channel expression [34], [35]. Increased CamKIIδ expression is moreover a signature feature of the phenotype switch in vascular smooth muscle [45], and recent work has identified CamKIIδ as a direct target of miR-145 [16]. Upregulation of CamKIIδ is thus an appealing candidate mechanism by which L-type Ca^2+^ channel expression drops in Dicer KO bladder. However, we did not detect a significantly altered expression of CamKIIδ in the Dicer KO bladder. We cannot rule out significant changes at earlier time points, but this finding suggests additional, or completely different, mechanisms. One possibility is that NFκB, which has been proposed to play a role for L-type Ca^2+^ channel expression in the vasculature [46], is involved. Additional work is required to test these possibilities.

The reduced scopolamine-sensitive (cholinergic) component of activation during electrical field stimulation in Dicer KO bladder is intriguing. This reduction was significantly more pronounced than was the reduction of contractility in response to carbachol. We interpret this to reflect impaired cholinergic neuro-effector transmission. Electron microscopy did not indicate an altered density of detrusor nerve terminals or overt changes in the size of the synaptic clefts, ruling out partial denervation. One remaining possibility is reduced release of acetylcholine from neural varicosities. Previous work has demonstrated important roles of miRNAs in neuronal differentiation and synaptic function [47], [48], but neuronal miRNAs are not targeted in our model so any pre-junctional effects must be indirect. Putative neuronal changes could, for example, depend on transfer (or loss thereof) of miRNAs from smooth muscle to neurons where proteins that play a role in synthesis and release of acetylcholine are targeted. A primary deficit in the smooth muscle cells, on the other hand, may involve secreted factors that signal to nearby neurons, matrix changes, or expression of acetylcholine esterase in the synaptic cleft. Whatever the case, it must be considered that a neurotransmission defect may in part be responsible for the reduced KCl responses. This is because depolarization is expected to cause release of transmitters and neuropeptides from peripheral nerve endings, including acetylcholine and neurokinins. Reduced neuronal release might therefore contribute less to KCl-induced force development in the KO bladder.

In summary, knockdown of miRNAs in the urinary bladder affects spontaneous micturition as well as contractile function of the isolated bladder. This is associated with reduced expression of contractile marker proteins and L-type Ca^2+^ channels. A reduction of the atropine/scopolamine-sensitive component of neurogenic activation together with an increased relative purinergic component has been reported for bladder instability [8], interstitial cystitis [9], and normal ageing [10]. The current study thus provides proof of principle that smooth muscle cells can be primary culprits in the chain of events that leads to detrusor instability as defined by the increased spontaneous micturition and the characteristic changes in neuro-effector transmission and contractility.

## Supporting Information

Figure S1 **Combined data on contraction induced by electrical field stimulation at 10 weeks post tamoxifen.** Data in Figure 4 A and B were merged in one panel to facilitate direct comparison of WT and KO data.
(TIF)

Figure S2 **Effect of Dicer deletion on electrical field stimulation-induced contraction at 5 weeks.** Full frequency response curves in control conditions, in the presence of scopolamine (1 mM), and after desensitization of purinergic receptors using α,β-methylene-ATP (10 mM) in the continued presence of scopolamine are shown for control and Dicer KO bladders in A and B, respectively, 5 weeks following Tamoxifen treatment. The cholinergic component of activation (C) was calculated by subtracting the force in the presence of scopolamine from force in control conditions. The purinergic component of activation (D) was calculated by subtracting residual force (after α,β-methylene-ATP and in the presence of scopolamine) from force in the presence of scopolamine. E shows the relative peak force on addition of α,β-methylene-ATP (n = 8–9).
(TIF)

Table S1 **MicroRNA (miRNA) qPCR-arrays define highly expressed miRNAs in the detrusor and demonstrate effective knock down of most miRNAs.** QPCR based miRNA arrays were run on pooled detrusor samples from control and smooth muscle-specific Dicer KO mice. Fold expression relative to housekeeping genes is shown in the middle column and the percentage of knockdown in Dicer KO bladders is shown in the right column. The expression levels shown are assuming equal efficiency of the primers. The data are from a single experiment from six pooled bladders of each genotype.
(TIF)
