# Supplementary figures and images for: Deletion of Dicer in Smooth Muscle Affects Voiding Pattern and Reduces Detrusor Contractility and Neuroeffector Transmission
Source: PLoS One. 2012 Apr 27;7(4):e35882. doi: 10.1371/journal.pone.0035882 (PMC3338793; doi:10.1371/journal.pone.0035882)

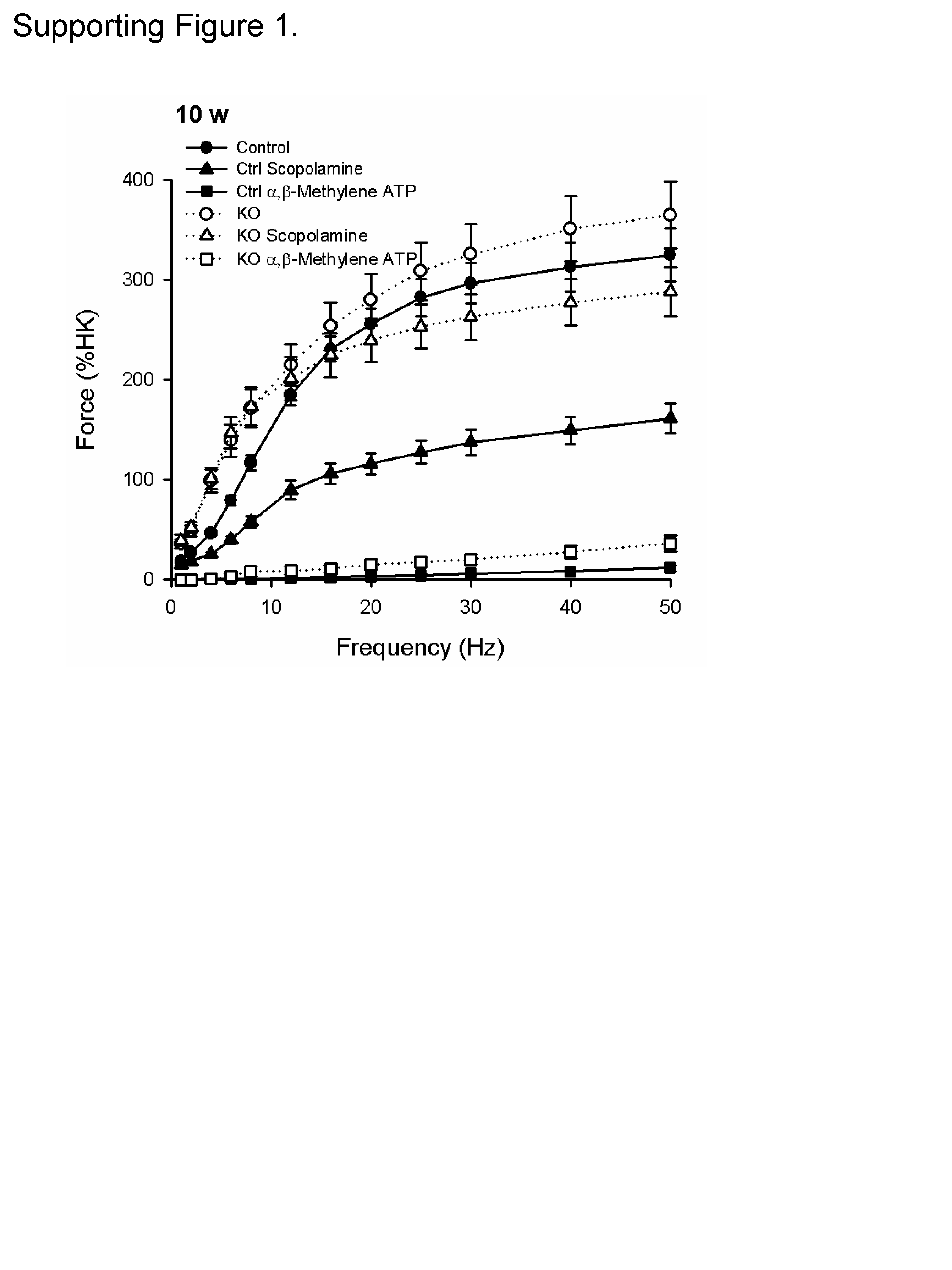

Supplement: Figure S1 — Combined data on contraction induced by electrical field stimulation at 10 weeks post tamoxifen. Data in Figure 4 A and B were merged in one panel to facilitate direct comparison of WT and KO data. (TIF) [file pone.0035882.s001.tif]

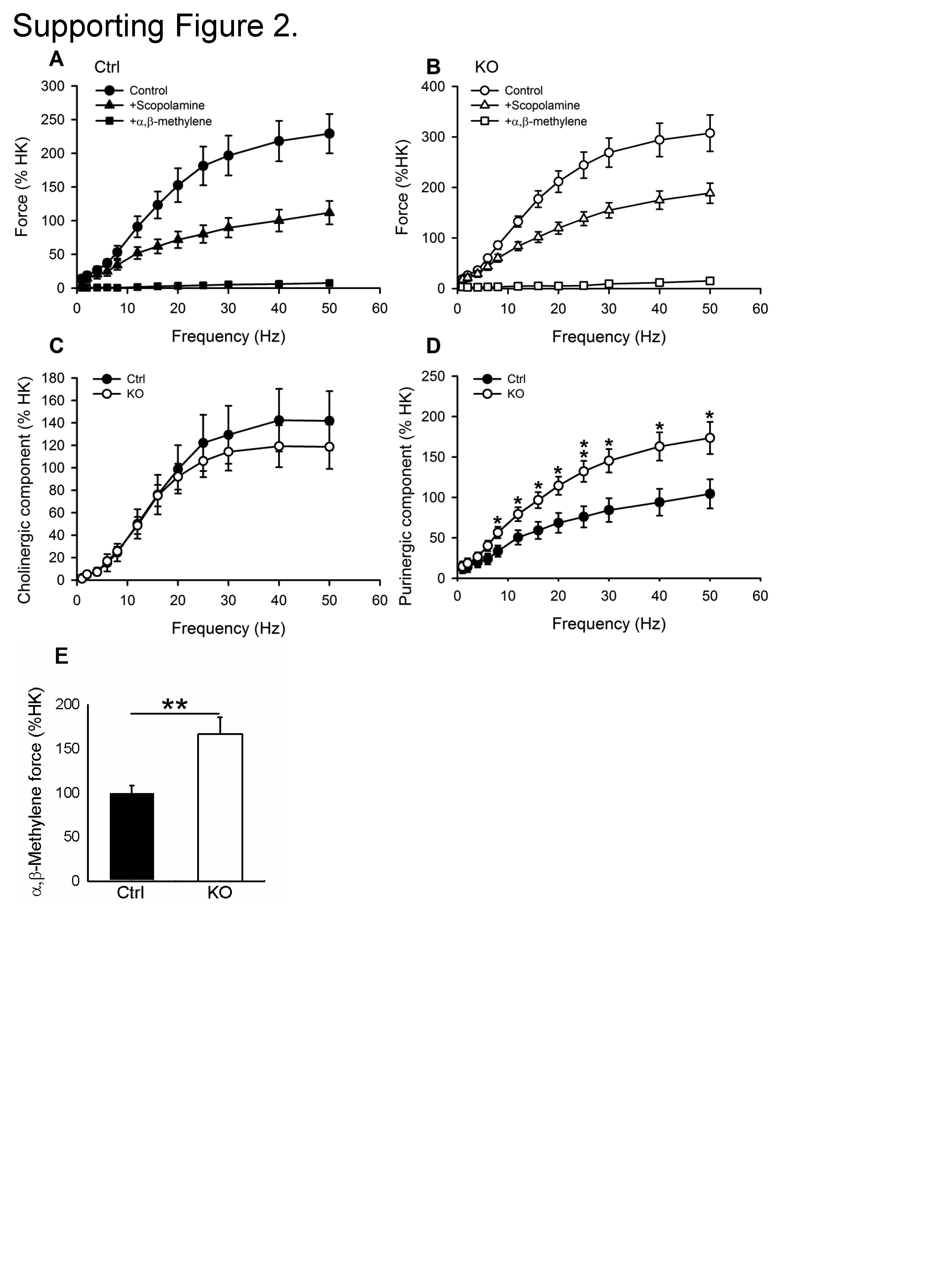

Supplement: Figure S2 — Effect of Dicer deletion on electrical field stimulation-induced contraction at 5 weeks. Full frequency response curves in control conditions, in the presence of scopolamine (1 mM), and after desensitization of purinergic receptors using α,β-methylene-ATP (10 mM) in the continued presence of scopolamine are shown for control and Dicer KO bladders in A and B, respectively, 5 weeks following Tamoxifen treatment. The cholinergic component of activation (C) was calculated by subtracting the force in the presence of scopolamine from force in control conditions. The purinergic component of activation (D) was calculated by subtracting residual force (after α,β-methylene-ATP and in the presence of scopolamine) from force in the presence of scopolamine. E shows the relative peak force on addition of α,β-methylene-ATP (n = 8–9). (TIF) [file pone.0035882.s002.tif]

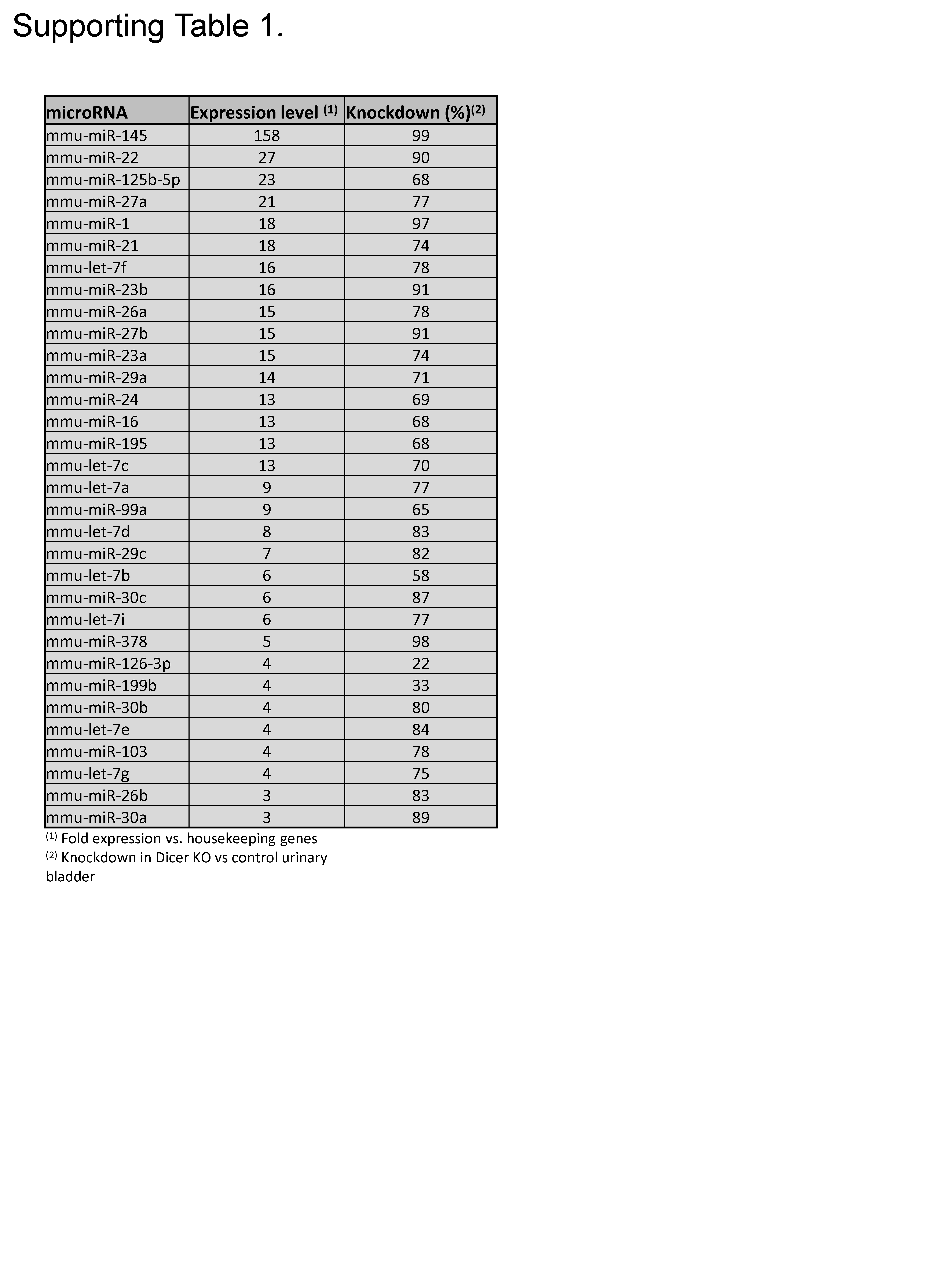

Supplement: Table S1 — MicroRNA (miRNA) qPCR-arrays define highly expressed miRNAs in the detrusor and demonstrate effective knock down of most miRNAs. QPCR based miRNA arrays were run on pooled detrusor samples from control and smooth muscle-specific Dicer KO mice. Fold expression relative to housekeeping genes is shown in the middle column and the percentage of knockdown in Dicer KO bladders is shown in the right column. The expression levels shown are assuming equal efficiency of the primers. The data are from a single experiment from six pooled bladders of each genotype. (TIF) [file pone.0035882.s003.tif]
